# WHEN RESILIENCE BECOMES RISK: PSYCHOSOCIAL RESOURCE PROFILES AND ALLOSTATIC LOAD AMONG AFRICAN AMERICAN MEN

**DOI:** 10.1093/geroni/igae098.1599

**Published:** 2024-12-31

**Authors:** Courtney Thomas Tobin, Angela Gutierrez, Christy Erving, Keith Norris, Roland Thorpe

**Affiliations:** University of California Los Angeles, Los Angeles, California, United States; Western University of Health Sciences, Pomona, California, United States; The University of Texas Austin, Austin, Texas, United States; University of California Los Angeles, Los Angeles, California, United States; Johns Hopkins University, Baltimore, Maryland, United States

## Abstract

There is a well-established link between psychosocial risks and psychological health among African American (AA) men. The psychosocial sources and physical health consequences of resilience (i.e., the ability to maintain good health despite adversity) remain underexplored. Using data from 283 AA men in the Nashville Stress and Health Study, the present study investigated the links between psychosocial resilience and allostatic load (AL), a biological indicator of physiological dysregulation. Latent class analysis (LCA) identified distinct resilience profiles comprised of eight psychosocial resources across four categories: coping strategies, sense of control, racial identity, and social support. Analysis of variance (ANOVA) tests determined significant class differences in men’s AL scores. LCA results confirm a four-class model was the best fit: Class 1 (high resources, 32%); Class 2 (high coping but low control, 13%); Class 3 (low resources but high racial identity, 20%); and Class 4 (low resources but high mastery, 34%). Results reveal lower AL (better health) among Classes 4 (m=0.31) and 1 (m=0.35), and higher AL among Classes 2 (m=0.44) and 3 (m=0.44). Findings indicate that the “quality” rather than the “quantity” of psychosocial resources matters for physical health among AA men, as positive health outcomes were observed among both low and high resource classes. Results suggest different resource combinations produce distinct patterns of resilience among AA men and underscore the need to further elucidate complex resilience processes among this population.

